# Supplementary material for: Impact of synonymous mutations in the blaTEM-3 gene on gene expression and Escherichia coli fitness
Source: Microbiol Spectr. 2025 Nov 18;14(1):e02695-25. doi: 10.1128/spectrum.02695-25 (PMC12772226; doi:10.1128/spectrum.02695-25)
Supplement: Supplemental figures — Fig. S1 and S2. [file spectrum.02695-25-s0001.pdf]

Impact of synonymous mutations in the *bla*$_{TEM-3}$ gene on gene expression and *Escherichia coli* fitness

Rinku Dhungana,[1,2] Heba Kaadan,[1] Aparna Paudel,[1] and Peter Oelschlaeger [1]

SUPPLEMENTAL MATERIALS

Index:

Page 2 FIG S1: Sequence alignment of *bla*$_{TEM-1a}$, *bla*$_{TEM-3}$(-sm), and *bla*$_{TEM-3}$(+sm) genes

Page 3 FIG S2: E-tests with cells expressing *bla*$_{TEM-3}$(-sm) and *bla*$_{TEM-3}$(+sm) genes

Page 4 References

```
blaTEM-3[+sm]      atgagtattcaacatttccgtgtcgcccttattcccttttttgcggcattttgccttcct    60
blaTEM-1a          atgagtattcaacatttccgtgtcgcccttattcccttttttgcggcattttgccttcct    60
blaTEM-3[-sm]      atgagtattcaacatttccgtgtcgcccttattcccttttttgcggcattttgccttcct    60
                   ************************************************************

blaTEM-3[+sm]      gtttttgctcacccagaaacgctggtgaaagtaaaagatgctgaagataagttgggtgca    120
blaTEM-1a          gtttttgctcacccagaaacgctggtgaaagtaaaagatgctgaagatcagttgggtgca    120
blaTEM-3[-sm]      gtttttgctcacccagaaacgctggtgaaagtaaaagatgctgaagataagttgggtgca    120
                   ************************************************ ***********

blaTEM-3[+sm]      cgagtgggttacatcgagctggatctcaacagcggtaagatccttgagagttttcgcccc    180
blaTEM-1a          cgagtgggttacatcgaactggatctcaacagcggtaagatccttgagagttttcgcccc    180
blaTEM-3[-sm]      cgagtgggttacatcgaactggatctcaacagcggtaagatccttgagagttttcgcccc    180
                   ***************** ******************************************

blaTEM-3[+sm]      gaagaacgttttccaatgatgagcacttttaaagttctgctatgtggtgcggtattatcc    240
blaTEM-1a          gaagaacgttttccaatgatgagcacttttaaagttctgctatgtggcgcggtattatcc    240
blaTEM-3[-sm]      gaagaacgttttccaatgatgagcacttttaaagttctgctatgtggcgcggtattatcc    240
                   *********************************************** ************

blaTEM-3[+sm]      cgtgttgacgccgggcaagagcaactcggtcgccgcatacactattctcagaatgacttg    300
blaTEM-1a          cgtgttgacgccgggcaagagcaactcggtcgccgcatacactattctcagaatgacttg    300
blaTEM-3[-sm]      cgtgttgacgccgggcaagagcaactcggtcgccgcatacactattctcagaatgacttg    300
                   ************************************************************

blaTEM-3[+sm]      gttaagtactcaccagtcacagaaaagcatcttacggatggcatgacagtaagagaatta    360
blaTEM-1a          gttgagtactcaccagtcacagaaaagcatcttacggatggcatgacagtaagagaatta    360
blaTEM-3[-sm]      gttaagtactcaccagtcacagaaaagcatcttacggatggcatgacagtaagagaatta    360
                   *** ********************************************************

blaTEM-3[+sm]      tgcagtgctgccataaccatgagtgataacactgcggccaacttacttctgacaacgatc    420
blaTEM-1a          tgcagtgctgccataaccatgagtgataacactgcggccaacttacttctgacaacgatc    420
blaTEM-3[-sm]      tgcagtgctgccataaccatgagtgataacactgcggccaacttacttctgacaacgatc    420
                   ************************************************************

blaTEM-3[+sm]      ggaggaccgaaggagctaaccgcttttttgcacaacatgggggatcatgtaaccgcctt    480
blaTEM-1a          ggaggaccgaaggagctaaccgcttttttgcacaacatgggggatcatgtaactcgcctt    480
blaTEM-3[-sm]      ggaggaccgaaggagctaaccgcttttttgcacaacatgggggatcatgtaactcgcctt    480
                   ****************************************************** ******

blaTEM-3[+sm]      gatcgttgggaaccggagctgaatgaagccataccaaacgacgagcgtgacaccacgatg    540
blaTEM-1a          gatcgttgggaaccggagctgaatgaagccataccaaacgacgagcgtgacaccacgatg    540
blaTEM-3[-sm]      gatcgttgggaaccggagctgaatgaagccataccaaacgacgagcgtgacaccacgatg    540
                   ************************************************************

blaTEM-3[+sm]      cctgcagcaatggcaacaacgttgcgcaaactattaactggcgaactacttactctagct    600
blaTEM-1a          cctgcagcaatggcaacaacgttgcgcaaactattaactggcgaactacttactctagct    600
blaTEM-3[-sm]      cctgcagcaatggcaacaacgttgcgcaaactattaactggcgaactacttactctagct    600
                   ************************************************************

blaTEM-3[+sm]      tcccggcaacaattaatagactggatggaggcggataaagttgcaggaccacttctgcgc    660
blaTEM-1a          tcccggcaacaattaatagactggatggaggcggataaagttgcaggaccacttctgcgc    660
blaTEM-3[-sm]      tcccggcaacaattaatagactggatggaggcggataaagttgcaggaccacttctgcgc    660
                   ************************************************************

blaTEM-3[+sm]      tcggcccttccggctggctggtttattgctgataaatctggagccagtgagcgtggatct    720
blaTEM-1a          tcggcccttccggctggctggtttattgctgataaatctggagccggtgagcgtgggtct    720
blaTEM-3[-sm]      tcggcccttccggctggctggtttattgctgataaatctggagccagtgagcgtgggtct    720
                   ********************************************** ********** ***

blaTEM-3[+sm]      cgcggtatcattgcagcactggggccagatggtaagccctcccgtatcgtagttatctac    780
blaTEM-1a          cgcggtatcattgcagcactggggccagatggtaagccctcccgtatcgtagttatctac    780
blaTEM-3[-sm]      cgcggtatcattgcagcactggggccagatggtaagccctcccgtatcgtagttatctac    780
                   ************************************************************

blaTEM-3[+sm]      acgacggggagtcaggcaactatggatgaacgaaatagacagatcgctgagataggtgcc    840
blaTEM-1a          acgacggggagtcaggcaactatggatgaacgaaatagacagatcgctgagataggtgcc    840
blaTEM-3[-sm]      acgacggggagtcaggcaactatggatgaacgaaatagacagatcgctgagataggtgcc    840
                   ************************************************************

blaTEM-3[+sm]      tcactgattaagcattggtaa     861
blaTEM-1a          tcactgattaagcattggtaa     861
blaTEM-3[-sm]      tcactgattaagcattggtaa     861
                   *********************
```

FIG S1. Multiple sequence alignment of the *bla*$_{\text{TEM-1a}}$, *bla*$_{\text{TEM-3}}$(-sm), and *bla*$_{\text{TEM-3}}$(+sm) genes prepared with CLUSTAL O (1.2.4). Three nonsynonymous mutations are highlighted in red. The c109a, g304a, and g706a mutations cause the Q39K, E104K, and G238S amino acid changes, respectively (amino acid numbering according to Ambler et al.(1)). Four synonymous mutations are highlighted in cyan.

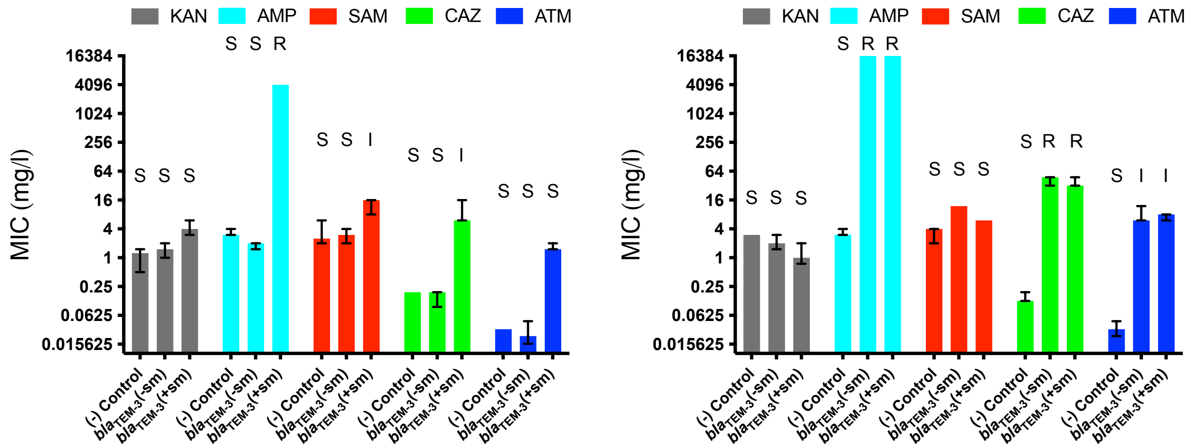

FIG S2. (A) E-Test results of "old" *E. coli* DH10B harboring pBC SK(+) vectors encoding the *bla*$_{\text{TEM-3}}$(-sm) or *bla*$_{\text{TEM-3}}$(+sm) genes or an empty vector ((-) Control). (B) Same as (A) except that cells were freshly transformed with vectors and selected with carbenicillin to ascertain full-length *bla*$_{\text{TEM-3}}$ genes. Bar lengths represent the median of three independent experiments, and error bars indicate the range of values. The phenotypes (S = susceptible; I = intermediate; R = resistant) according to the E-Test strip supplier (Liofilchem®) Interpretive Criteria according to the CLSI (2) are shown above the bars. The values of AMP R were beyond the E-Test strip range and were determined on agar plates. The AMP values determined in this way in panel (B) were >8,192 mg/l and are displayed as 16,386 mg/l.

References

1. Ambler RP, Coulson AF, Frere JM, Ghuysen JM, Joris B, Forsman M, Levesque RC, Tiraby G, Waley SG. 1991. A standard numbering scheme for the class A beta-lactamases. Biochem J 276 (Pt 1)**:**269-270. http://dx.doi.org/10.1042/bj2760269.
2. Clinical and Laboratory Standards Institute. 2024. Performance Standards for Antimicrobial Susceptibiity Testing; 34th ed. CLSI supplement M100S, Wayne, PA.
